# Mild Cognitive Impairment in *de novo* Parkinson's Disease: Selective Attention Deficit as Early Sign of Neurocognitive Decay

**DOI:** 10.3389/fpsyg.2021.546476

**Published:** 2021-03-30

**Authors:** Davide Maria Cammisuli, Cristina Pagni, Giovanni Palermo, Daniela Frosini, Joyce Bonaccorsi, Claudia Radicchi, Simona Cintoli, Luca Tommasini, Gloria Tognoni, Roberto Ceravolo, Ubaldo Bonuccelli

**Affiliations:** ^1^Department of Clinical and Experimental Medicine, University of Pisa, Pisa, Italy; ^2^Azienda Unità Sanitaria Locale (USL) Toscana Nord Ovest, Pisa, Italy; ^3^Institute of Neuroscience, National Research Council (CNR), Pisa, Italy; ^4^Department of Neurosciences, Psychology, Drugs and Child Health Area, School of Psychology, University of Florence, Florence, Italy

**Keywords:** mild cognitive impairment, caudate nucleus, visual search, selective attention, *de novo* Parkinson's disease

## Abstract

**Background:** In the present study, we aimed to better investigate attention system profile of Parkinson's disease-Mild Cognitive Impairment (PD-MCI) patients and to determine if specific attentional deficits are associated with 123I-FP-CIT SPECT.

**Methods:** A total of 44 de novo drug-naïve PD patients [(27) with normal cognition (PD-NC) and 17 with MCI (PD-MCI)], 23 MCI patients and 23 individuals with subjective cognitive impairment (SCI) were recruited at the Clinical Neurology Unit of Santa Chiara hospital (Pisa University Medical School, Italy). They were assessed by a wide neuropsychological battery, including Visual Search Test (VST) measuring selective attention. Performances among groups were compared by non-parametric tests (i.e., Kruskal-Wallis and Mann-Whitney, Bonferroni corrected). Further, Spearman's rank correlations were performed to explore the association between neuropsychological variables and 123I-FP-CIT SPECT data in PD subgroup.

**Results:** PD-MCI patients performed worse on VST than patients with PD-NC (*p* = 0.002), patients with MCI and individuals with SCI (*p* < 0.001). The performance of PD-MCI patients on VST significantly correlated with caudate nucleus 123I-FP-CIT SPECT uptake (rho = 0.582, *p* < 0.05), whereas a negative correlation between such test and 123I-FP-CIT SPECT uptake in the left putamen (rho = −0.529, *p* < 0.05) was found in PD-NC patients.

**Conclusions:** We suggest that selective attention deficit might be a trigger of cognitive decay in de novo PD-MCI patients. The VST should be routinely used to detect attentional deficits in hospital clinical practice, in the light of its closely association with dopamine depletion of basal ganglia in mildly impaired PD patients.

## Background

Analogous to the construct of mild cognitive impairment (i.e., MCI) in Alzheimer's disease (AD) (Petersen et al., 2001; Winblad et al., 2004), Parkinson's disease–mild cognitive impairment (PD-MCI) identifies an earliest clinically recognizable phase of PD dementia (PDD) (Hoogland et al., 2018). Cognitive decline is present in ~30–40% of PD patients (Wojtala et al., 2019). Neuropsychological deficits can be detected even in untreated patients and usually worsen with disease progression and affect Quality of Life (QoL) of patients (Li et al., 2019). PD-MCI patients with deficits in visuospatial skills and frontal/executive functions are more likely to convert into PDD (Vasconcellos et al., 2019). Furthermore, previous studies have reported that a great amount of PD patients (nearly 80%) with MCI develop dementia in the course of the disorder (Li et al., 2019). Identification of prodromal stages is thus relevant, so that therapeutic interventions and rehabilitation strategies can be administered at a time when they are most effective (Cammisuli et al., 2020).

Selective attention constitutes an intrinsic component of perceptual representation system and it is hierarchically organized. Two attentional models are recognized to explain cognitive processing and perceptual selection of visual stimuli: one is a bottom-up model that is involuntary and stimulus-driven, depending on its salience over the environment; the other one is a top-down model, depending on goal-directed behavior (Beck and Kastner, 2005). While neurons in the earliest level of the visual system (i.e., lateral geniculate nucleus) are more influenced by properties of visual scenes and retinal image, neurons in the prefrontal and parietal cortex are more likely to be driven by behavioral goals (Yantis, 2008). Several brain regions have been investigated for their contribution to visual selective attention, including posterior parietal cortex and prefrontal cortex sub-regions (i.e., frontal eye field and the supplementary eye field) as well as the superior colliculus, which is relevant for eye movement (Yantis, 2008). Particularly, a dorsal attention network including intraparietal sulci and frontal eye fields has been recently brought into play also for describing selective attention modulation on brain circuits (Rosembaum et al., 2018).

On one hand, even if memory impairment is considered the most relevant hallmark in MCI, attentional problems have also been identified during the preclinical phase of AD and some studies have also indicated that attention control deficit and compromised set-shifting abilities are among the early signs in individuals with subjective cognitive impairment (SCI) (Tu et al., 2018). On the other hand, neuropsychological deficits of attention system are well-known in Parkinson's disease (PD) literature, too. A frontal regulation disturbance of attention in PD *due to* the degeneration of dopaminergic mesocortical innervation of the prefrontal cortex has been recognized very early by researchers (Stam et al., 1993). PD patients also show impairment in attentional shifting and orienting network involving the selection of information from sensory inputs associated with the activity of temporal parietal junction, superior parietal lobe, and frontal eye fields (Zhou et al., 2012). Particularly, some studies have shown different deficits of attention system also in PD-MCI patients mainly pertaining divided attention, sensitivity to interference, and visual processing speed (Possin et al., 2008; Wen et al., 2017; Bezdicek et al., 2018; Weil et al., 2018). However, little is known about the specific role of visual selective attention in non-demented PD patients and its relation with functional neuroimaging to date. Our study aimed at evaluating visual search ability in *de novo* PD-MCI patients and exploring the association with ^123^I-FP-CIT SPECT binding values.

## Methods

### Participants

A total of 108 participants were discontinuously enrolled at the Neurology outpatient clinic at Santa Chiara Hospital, Pisa University. They consisted of 23 individuals with SCI, 23 patients with MCI, and 44 patients with *de novo* PD. Diagnosis was given according to the criteria of Jessen et al. (2014), Winblad et al. (2004), and Gelb et al. (1999) for SCI, MCI, and PD, respectively. Further, *de novo* drug naïve PD patients were divided into two groups, on the basis of clinical diagnosis and neuropsychological assessment, resulting in 27 patients with normal cognition (i.e., PD-NC) and 17 patients with PD-MCI, according to Litvan et al. (2012) criteria at Level I. Exclusion criteria encompassed the following ones: (i) patients with atypical signs or symptoms suggesting other causes for parkinsonism (i.e., vascular and iatrogenic parkinsonism or Parkinson plus syndromes); (ii) anxiety and depression symptoms, as measured by scores above 18 at the Hamilton Anxiety Rating Scale (Hamilton, 1959) and at the Hamilton Depression Rating Scale (Hamilton, 1960), respectively; (iii) history of other neurological diseases (e.g., epilepsy, stroke, traumatic brain injury, etc.); (iv) patients fulfilling criteria for Parkinson's disease dementia (PDD) (Emre et al., 2007); (v) cardiovascular, metabolic, or psychiatric syndromes; and (vi) any other medical condition that may significantly interfere with cognitive status.

### PD Patients' Clinical Assessment

Both PD-NC and PD-MCI were newly diagnosed *drug-naïve* patients with a disease duration <2 years. They were at their first assessment in a University Hospital-based Movement Disorder Unit. The patients underwent a ^123^ioflupane-fluoropropyl-carbomethoxy-3-beta-4-iodophenyltropane single photon emission computed tomography (^123^I-FP-CIT SPECT) to confirm PD diagnosis and brain magnetic resonance imaging (MRI) or computed tomography (CT) in the case that MRI was unfeasible to rule out other brain diseases. They were comprehensively assessed at baseline by means of the Unified Parkinson's Disease Rating Scale Part III (UPDRS-III) to assess motor symptoms (Movement Disorder Society Task Force on Rating Scales for Parkinson's Disease, 2003) and by the Beck Depression Inventory (BDI) (Beck et al., 1988) to rate depression. Subgroups of PD that include tremor dominant (AT), rigido-akinetic, mixed, and postural instability-gait disorder (PIGD) were classified by means of the UPDRS III, by using a method similar to that used by Lewis et al. (2005).

### Brain SPECT

All the patients underwent brain SPECT with ^123^I-FP-CIT (DaTSCAN®, GE Healthcare, UK), which was injected intravenously at a dose of ~185 MBq, preceded by thyroid blockade according to the standard procedure. Scans were acquired between 3 and 4 h after tracer injection. The patients were scanned with a dual-head gamma camera (Discovery 710, GE Healthcare, Milwaukee, WI, USA) equipped with high-resolution low-energy parallel hole collimators. The acquisition parameters were as follows: circular orbit over 360°, 120 projections (angular sampling 3°), matrix 128 × 128, pixel size 3 mm, and overall scanning time of 35–40 min. SPECT reconstruction was performed using the ordered subset expectation maximization (OSEM) algorithm (2 iterations and 10 subsets) and by applying a 3D post-reconstruction filter (Butterworth, order 10, cut-off 0.5 cycles/cm) and attenuation correction (Chang method, μ = 0.12 cm^−1^). Reconstructed images in DICOM format were semi-quantitatively analyzed with the BasGan V2 software 20 that allows automatic, 3-D segmentation of caudate nucleus and putamen in each hemisphere by means of a high-definition, 3-D striatal template, derived from Talairach's atlas, and generates a 3-D occipital ROI for background evaluation. Moreover, the software includes partial volume effect correction in the process of binding computation of the caudate nucleus, putamen, and background. Caudate nucleus and putamen ^123^I-FP-CIT binding was subtracted by background as follows [(caudate nucleus or putamen binding–background binding)/background binding] in order to obtain specific to non-displaceable binding ratios of the caudate nucleus and the putamen of each hemisphere.

### Neuropsychological Assessment

After neuroimaging, PD patients underwent the neurocognitive evaluation in the following week. All the participants were assessed by a neuropsychological battery, including Mini Mental State Examination (MMSE) (Magni et al., 1996); Memory span (Orsini et al., 1987); Rey Auditory Verbal Learning Test (RAVLT) immediate and delayed recall (Carlesimo et al., 1996); Rey Osterrieth Complex Figure (ROCF) copy, immediate, and delayed reproduction (Carlesimo et al., 1996); Stroop Test (Caffarra et al., 2002); Phonemic Fluency test (Carlesimo et al., 1996); and Visual Search Test (VST) (Spinnler and Tognoni, 1987).

Specifically, in the VST, the examinee is required to visualize three digit matrices, each consisting of 13 lines and 10 numbers (from 0 to 9) randomly located. He/she is asked to bar the number/s equal to those printed on the top of the matrices (i.e., 5 for the first one; 2 and 6 for the second one; 1, 4, and 9 for the third one). The maximum time to complete each task is 45 s, and the number of corrected barred items is calculated as a raw score.

### Statistical Analysis

Raw scores of the neuropsychological tests were transformed into adjusted age and education scores. A comparison of demographic characteristics was performed using the Kruskal–Wallis for non-Gaussian distributions. The distribution of our collected neuropsychological data did not pass the Kolmogorov-Smirnov Test. Thus, non-parametric statistics (i.e., Kruskal–Wallis and Mann–Whitney *U* test) were used to compare subgroup performances on the neuropsychological measures. A *p* < 0.05 (Bonferroni corrected) was set to reach significance level. The effect size was calculated by *r* (cf. Vargha and Delaney, 2000: 0.10– <0.30 = small effect; 0.30– <0.50 = medium effect; ≥0.50 = large effect).

Spearman's rank correlations were then performed to further investigate the association between neuropsychological variables and ^123^I-FP-CIT SPECT binding values as well as UPDRS-III and VST. The Statistical Package for the Social Sciences (SPSS) 23.0 software (SPSS Inc., Chicago, IL) was used to perform data analysis.

## Results

Descriptive statistics (*median values*) of the groups on neuropsychological measures and of the PD subgroups on clinical parameters were first reported (Tables 1, 2). The groups were well-matched in terms of socio-demographic variables of age and education (*p* > 0.05). Furthermore, PD-MCI and PD-NC did not significantly differ in terms of disease duration, UPDRS-III scores, and motor phenotypes, except for axial score (*p* < 0.05), side predominantly affected at the onset, and striatal binding values of ^123^I-FP-CIT SPECT (*p* > 0.05).

**Table 1:** Descriptive statistics (median values) of the four groups on neuropsychological tests.

	**SCI *(n = 32) median***	**MCI *(n = 32) median***	**PD-NC *(n = 27) median***	**PD-MCI *(n = 17) median***
MMSE	27.7	26.2	26.4	25.7
Memory Span	10	9	10.0	8.0
RAVLT—IR	43.5	30	38.25	30
RAVLT—DL	8.9	3.8	7.8	6.4
ROCF—C	32.65	31.5	31.3	25.8
ROCF—IR	18.5	11.4	14.9	10.9
ROCF—DL	17.7	10.9	15.4	11.8
VST	52.12	46.5	30.6	25.9
ST Interference/Time	11.6	19.6	15.2	23.6
ST Interference/Error	−0.7	0	−0.5	0.1
Phonemic Fluency	30.9	31.4	32.4	27.1

*MMSE, Mini Mental State Examination; RAVLT—IR, Rey Auditory Verbal Learning Test—Immediate Recall; RAVLT—DL, Rey Auditory Verbal Learning Test—Delayed Recall; ROCF—C, Rey Osterrieth Complex Figure—Copy; ROCF—IR, Rey Osterrieth Complex Figure—Immediate Reproduction; ROCF—DL, Rey Osterrieth Complex Figure—Delayed Reproduction; VST, Visual Search Test; ST, Stroop Test; BDI, Beck Depression Inventory; UPDRS-III, Unified Parkinson's Disease Rating Scale-Part III; RCN, right caudate nucleus; MCN, medium caudate nucleus; LCN, left caudate nucleus; RP, right putamen; MP, medium putamen; LP, left putamen*.

**Table 2:** Descriptive statistics (median values) of the PD subgroups on clinical indexes.

	**PD-NC 4 *(n = 27) median***	**PD-MCI 5 *(n = 17) median***
BDI	2.0	2.0
UPDRS-III	14	21
Tremor score	0.2	0.4
Brady score	0.5	0.7
Rigidity score	0.6	0.8
Axial score	0.2	0.6
I-FP-CIT DaTScan RCN	3.2	2.9
I-FP-CIT DaTScan MCN	3.2	2.9
I-FP-CIT DaTScan LCN	3.1	2.9
I-FP-CIT DatScan RP	2.4	2.1
I-FP-CIT DatScan MP	2.4	2.9
I-FP-CIT DaTScan LP	2.4	2.3

*BDI, Beck Depression Inventory; UPDRS-III, Unified Parkinson's Disease Rating Scale—Part III; RCN, right caudate nucleus; MCN, medium caudate nucleus; LCN, left caudate nucleus; RP, right putamen; MP, medium putamen; LP, left putamen*.

**Figure 1:**
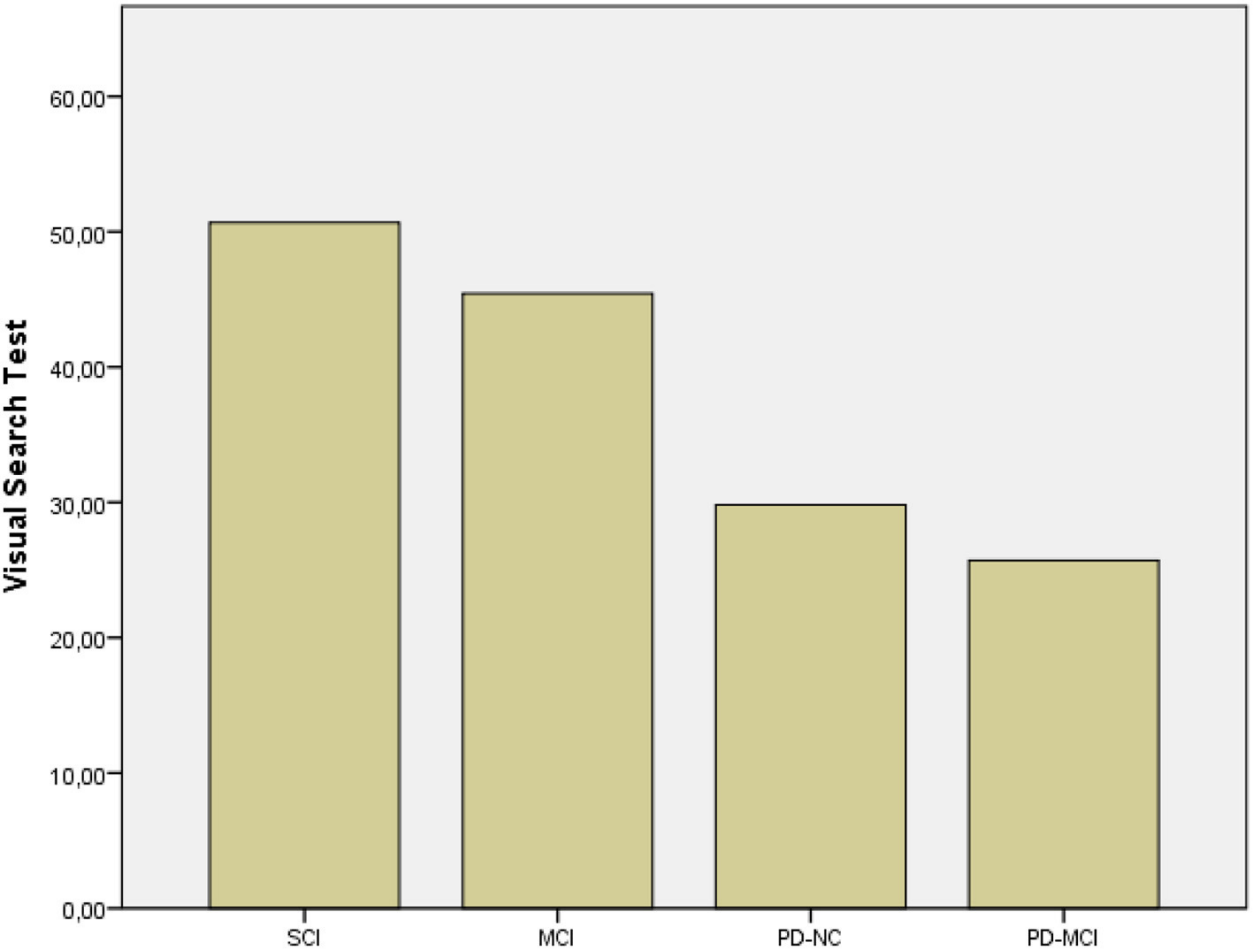
Results of subgroups on VST. SCI, Subjective Cognitive Impairment; MCI, Mild Cognitive Impairment; PD-NC, Parkinson's Disease-Normal Cognition; PD-MCI, Parkinson's Disease-Mild Cognitive Impairment.

The Kruskal–Wallis revealed significant differences between groups' performances on MMSE (*p* < 0.001), RAVLT immediate and delayed recall (*p* < 0.001), VST (*p* < 0.001), and ROCF immediate (*p* < 0.001) and delayed recall (*p* < 0.001). *Post hoc* comparison by the Mann–Whitney indicated that both PD-MCI and MCI patients obtained lower scores on MMSE than individuals with SCI (*p* < 0.001 and *p* = 0.002, respectively), as expected. PD-MCI patients perform worse on VST (*p* < 0.001, *r* = 0.808) than patients with MCI (Figure 1) that, in turn, report lower scores on RAVLT delayed recall (*p* < 0.001, *r* = 0.585) than PD-MCI patients. These results were further enhanced by the fact that PD-MCI patients obtained lower scores than individuals with SCI (*p* < 0.001, *r* = 0.835) and PD-NC (*p* = 0.002, *r* = 0.397) on VST (Figure 1) and MCI patients obtained lower scores on RAVLT immediate and delayed recall than individuals with SCI (*p* < 0.001, *r* = 0.770; *p* < 0.00, *r* = 0.820) and on RAVLT delayed recall than patients with PD-NC (*p* < 0.001, *r* = 0.704). Furthermore, MCI patients performed worse than individuals with SCI on ROCF immediate and delayed recall (*p* < 0.001, *r* = *0*.552; *p* < 0.001, *r* = 0.631).

**Figure 2:**
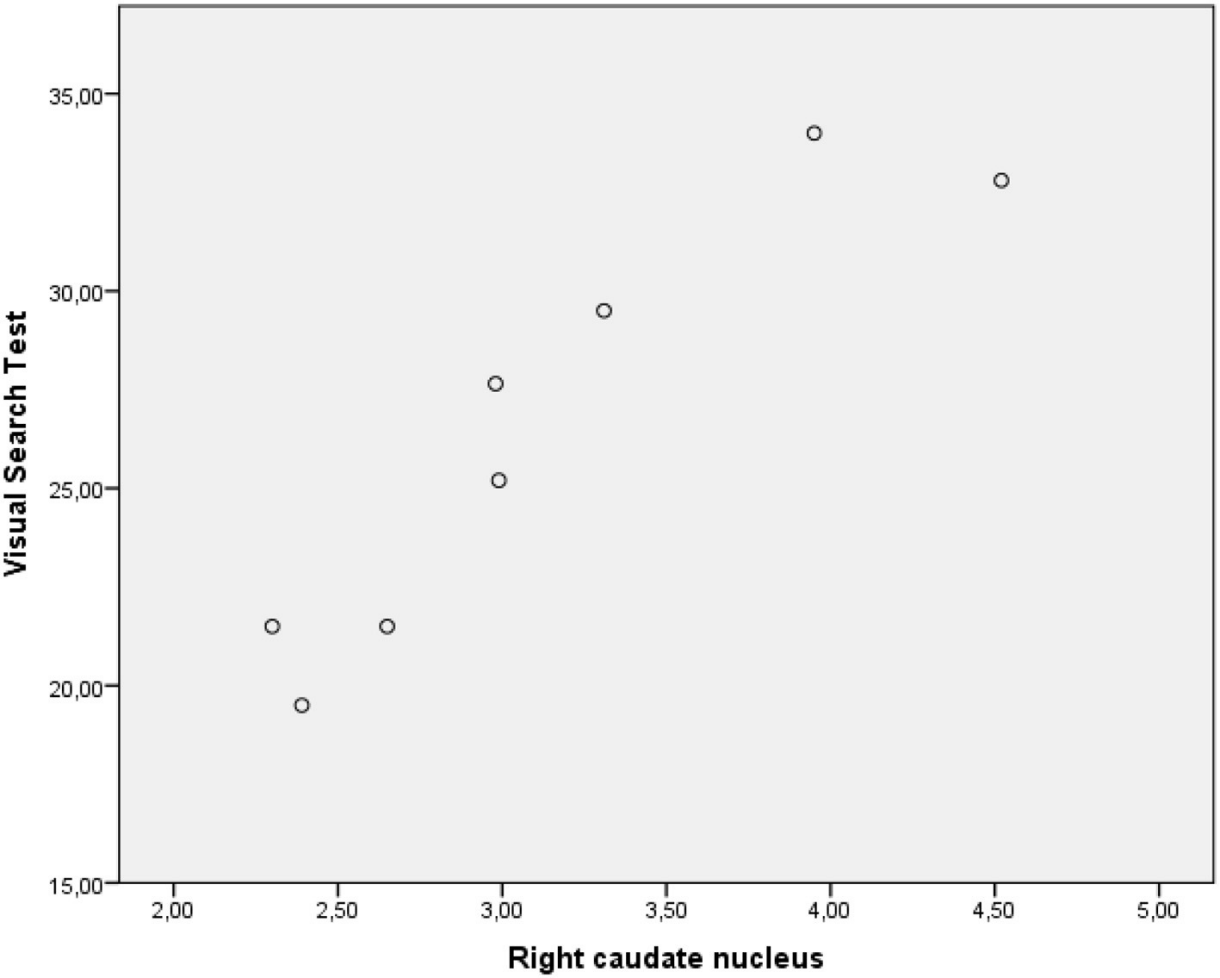
Graphic representation of correlation between VST scores and 123I-FP-CIT binding in the right caudate nucleus (PD-MCI).

**Figure 3:**
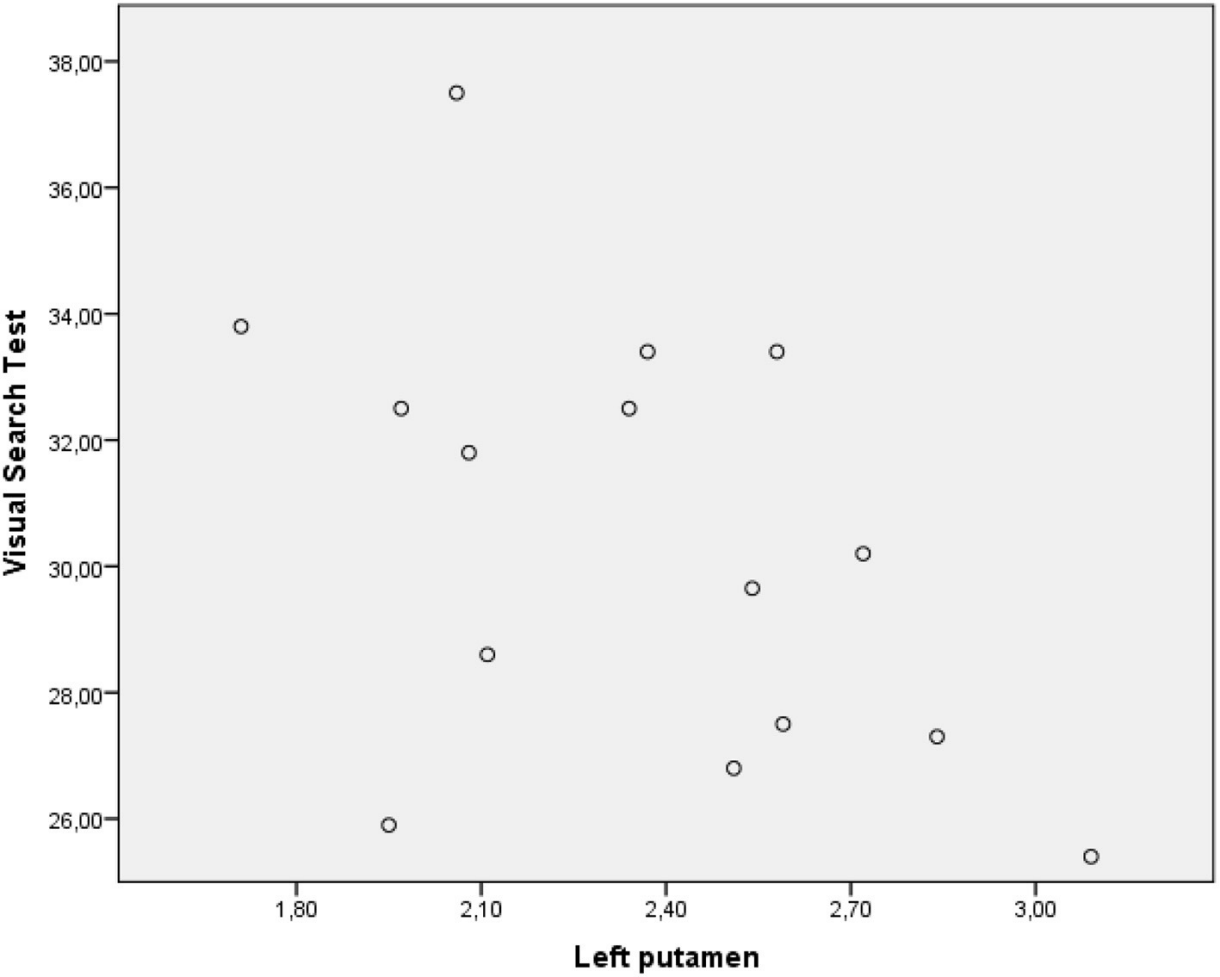
Graphic representation of correlation between VST scores and 123I-FP-CIT binding in the left putamen (PD-NC).

The performance of PD-MCI patients on VST significantly correlated with ^123^I-FP-CIT binding in the right caudate nucleus (*r*s =0.582, *p* < 0.05), whereas the performance of patients with PD-NC negatively correlated with dopamine transporter (DAT) availability in the left putamen (*r*s = −0.529, *p* < 0.05) (Figures 2, 3). Any other neuropsychological measure in both PD-MCI and PD-NC correlated with striatal DAT binding. UPDRS-III scores of PD-MCI patients were negatively associated with VST scores (*r*s = −0.783, *p* < 0.01) while no significant correlation was found for PD-NC.

## Discussion

To the best of our knowledge, this is the first paper detecting a specific deficit of selective attention in *de novo* PD-MCI that is significantly associated with the right caudate DAT binding. Remarkably, the test used to detect such an attentional deficit (i.e., VST) requires specific aspects of visual processing including signal detention, psychomotor speed, visuospatial working memory, and attentional control (Barletta-Rodolfi et al., 2011). Particularly, one aspect, i.e., psychomotor speed, was influenced by motor impairment of PD-MCI patients. If some attentional deficits in PD such as impaired vigilance with alertness level fluctuation and subtle visuospatial and perceptive impairments (i.e., difficulties with extra personal space perception and objects shape recognizing) seem to be linked to acetylcholine, noradrenaline, and serotonin (Biundo et al., 2016), we pointed out that the observed selective attention deficit was associated with dopamine depletion at the caudate level.

Changes in dopaminergic availability are known to affect fronto-striatal networks in PD, and dopamine medication aiming at counteracting motor dysfunction may also ameliorate frontal/executive abilities at a certain level (cf. Biundo et al., 2016). Our findings are in line with the previous studies on *de novo* PD patients of Siepel and Bronnick (2014) using the same neuroimaging technique that found a positive association between total striatal dopamine transporter binding and attention/executive deficits (but not with memory or visuospatial ones) and of Chung et al. (2018) using ^18^F-FP-CIT PET scan, concluding that DAT availability in the caudate is an important determinant of PD-related cognitive impairment able to discriminate MCI from normal cognition. However, neither Siepel and Bronnick (2014) nor Chung et al. (2018) used a visual selective attention test such as the VST.

Our results also outlined the pivotal role played by the caudate nucleus in visual attention, given that PD-MCI patients' performance on the VST was directly associated with ^123^I-FP-CIT binding of such brain area conversely to PD-NC patients presenting a negative correlation with the other part of the *striatum* (i.e., putamen). As evidenced by an outstanding literature review (Seger, 2013), visual attention involves, collects, and integrates sensory and cognitive data about the environment, in order to focus on potentially important targets and their spatial location: frontoparietal regions involved in visual attention including frontal eye fields and parietal cortex directly interact with the caudate.

The other neuropsychological findings are consistent with the expectations for which MCI patients reported a more severe episodic memory impairment both on verbal (i.e., RAVLT delayed recall) and visuospatial tests (i.e., ROCF immediate and delayed reproduction) than the other groups. Deficits of word list recall are also widely recognized in MCI patients Bennett et al., 2006, representing a prodromal stage of AD-related deterioration, associated with atrophy of medial temporal lobe (Andrés et al., 2019; Parnetti et al., 2019). Our findings are consistent with the literature, indicating that memory deficits of MCI patients are specifically reported on ROCF immediate and delayed reproduction tasks (Takayama, 2010) whereas PD-MCI patients' performances on ROCF are particularly revealed on copy task, which implies planning functions related to frontal domains (Biundo et al., 2013, 2014).

In this study, we documented the presence of a specific cognitive disorder of selective attention in *de novo* PD-MCI patients that may be—*in part*—explained in accordance to psychomotor speed reduction, as a cognitive ability related to the VST. In line with the fact that the level of disruption in visuospatial attentional functions increases with disease severity (cf. Mari et al., 1997), we would stress that such a deficit might represent a trigger of cognitive decay, given that it is not present in patients with PD-NC. Selective attention deterioration is associated with dysfunction in caudate nucleus and the decrease of dopaminergic projections of the brainstem toward cortical area and basal ganglia. We suggest to use the VST in detecting attentional deficits for routinely neuropsychological assessment of PD patients, in the light of its close association with dopamine depletion in basal ganglia of mildly impaired ones. Some studies using eye tracking techniques (Rose et al., 2013; Rosa et al., 2017) that can record eye movements and other measures, which lead to a higher degree of comprehension on cognitive processes associated with attention in visual tasks, may be used as complementary techniques to better understand neural processes underlying attention in *de novo* PD-MCI patients. However, our findings should be read cautiously by psychologists working in clinical settings, given that neuropsychological tests measuring attention system abilities usually contemplate more than one process (i.e., *the task impurity problem*) and attentional processes *themselves* could not be strictly evaluated in isolation (cf. Strauss et al., 2006).

Our study provides further evidence that PD-MCI requires specific psychodiagnostic tools for diagnosis and disease progression monitoring. To date, the number of studies comparing PD-MCI patients with MCI patients and even individuals with SCI is actually too small. This represents a strength of our investigation. In comparison to the previous investigations (Pistacchi et al., 2015; Besser et al., 2016; Hessen et al., 2016), we would stress that our research represents a step forward in specifying the attention system profile of *de novo* PD-MCI patients. However, our study tested a limited sample. Thus, it should be implemented by the collection of more extensive data—even prospectively, especially for PD-NC—to allow researchers to infer more robust conclusions and generalizability of the findings. Another constraint of our study was that PD-MCI was not classified by using Level II assessment of Litvan et al.' criteria (2012). A wider neuropsychological battery including at least two tests for each cognitive domain (i.e., attention/working memory, language, executive functioning, memory, and visuospatial skills) might bring out other typical neurocognitive defects potentially linked to basal ganglia dopamine reduction (e.g., frontal-executive ones). Future research should also contemplate comparison studies between *de novo* vs. pharmacological treated PD-MCI patients, in order to analyze such a visual selective attention deterioration at its best.

Impaired attention is a significant determinant of QoL in PD (Lawson et al., 2016). Cognitive rehabilitation interventions provided by the Attention Processing Training (APT) computerized program should be used to ameliorate sustained, divided, alternating, and selective attention of non-demented PD patients (cf. Mohlman et al., 2011), in order to delay a more severe cognitive deterioration.

## Data Availability Statement

Publicly available datasets were analyzed in this study. This data can be found here: https://etd.adm.unipi.it/t/etd-06262018-225446/.

## Ethics Statement

The studies involving human participants were reviewed and approved by Azienda Ospedaliero-Universitaria Pisana, Dipartimento Area Amministrativa- U.O. AFFARI GENERALI SEGRETERIA AMMINISTRATIVA DEL CEAVNO, 67, Via Roma, 56126 PISA—Tel. +39-050-996111. The patients/participants provided their written informed consent to participate in this study.

## Author Contributions

DMC made the most substantial contribution in conceiving the original ideal, performing data analysis, and writing the manuscript. CP and GT significantly contributed in research design planning and supervision of dataset. JB, RC, SC, and LT assessed patients at the Neurology Clinic by neuropsychological tests. DF made the neurological examination of patients with Parkinson's disease patients at the Neurology Clinic. GP gave support in explaining the association between neuropsychological data and brain imaging results as well as in contributing to the final version of the manuscript. RC and UB revised the manuscript for intellectual content. All authors contributed to the article and approved the submitted version.

## Conflict of Interest

The authors declare that the research was conducted in the absence of any commercial or financial relationships that could be construed as a potential conflict of interest.
